# Are changes in the urinary sodium-to-potassium ratio associated with changes in blood pressure in a healthy population with low urinary sodium-to-potassium ratios? Eight-year follow-up results from the KOBE Study

**DOI:** 10.1038/s41440-026-02621-9

**Published:** 2026-04-13

**Authors:** Mizuki Kawahara, Rumi Tsukinoki, Naomi Miyamatsu, Kazuyo Kuwabara, Sachimi Kubo, Yoshimi Kubota, Aya Higashiyama, Aya Hirata, Takumi Hirata, Junji Miyazaki, Daisuke Sugiyama, Yoshihiro Miyamoto, Tomonori Okamura

**Affiliations:** 1 Department of Public Health Nursing, Institute of Science Tokyo, Tokyo, Japan; 2 Department of Clinical Nursing, Shiga University of Medical Science, Shiga, Japan; 3 Department of Preventive Medicine and Public Health, Keio University School of Medicine, Tokyo, Japan; 4 Department of Public Health, Kindai University Faculty of Medicine, Osaka, Japan; 5 Department of Preventive Medicine, School of Medicine, Hyogo Medical University, Hyogo, Japan; 6 Department of Hygiene, Wakayama Medical University, Wakayama, Japan; 7 Human Care Research Team, Tokyo Metropolitan Institute for Geriatrics and Gerontology, Tokyo, Japan; 8 Division of Public Health, Department of Social Medicine, Graduate School of Medicine, The University of Osaka, Osaka, Japan; 9 Faculty of Nursing and Medical Care, Keio University, Kanagawa, Japan; 10 Open Innovation Center, National Cerebral and Cardiovascular Center, Osaka, Japan; 11 Center for Cluster Development and Coordination, Foundation for Biomedical Research and Innovation, Hyogo, Japan

**Keywords:** Blood pressure, Urinary sodium-to-potassium (Na/K) ratio, Implementation hypertension, Digital hypertension

## Abstract

Little is known about the associations between changes in the urinary sodium-to-potassium (Na/K) ratio and blood pressure (BP) in healthy individuals. Using survey data, urinary data, and BP data from the KOBE Study, this longitudinal study aimed to assess the associations between changes in the urinary Na/K ratio and BP in a healthy Japanese population over an 8-year follow-up period. We analyzed 567 participants aged 40–74 years who did not initiate antihypertensive or cardiovascular disease treatment during follow-up. Changes in the spot urinary Na/K ratio and BP were calculated by subtracting the baseline values from the follow-up measurements, and their associations were examined using a multivariable linear regression analysis that adjusted for sex, age, body mass index, baseline Na/K ratio and BP, low-density lipoprotein, hemoglobin A1c, ethanol intake, smoking status, salt taste sensitivity, years of education, employment status, baseline survey season, and 8-year follow-up survey season. The study cohort had a mean spot urinary Na/K ratio of 2.1 at baseline. While BP increased significantly over the 8-year follow-up period, the urinary Na/K ratio remained unchanged. However, urinary Na/K ratio changes were positively associated with changes in systolic BP (β = 1.48, *p* = 0.001) and diastolic BP (β = 0.76, *p* = 0.004). In conclusion, increases in spot urinary Na/K ratios were predictive of long-term BP elevation in a healthy, normotensive population with near-optimal urinary Na/K ratios at baseline. These findings indicate that maintaining a low urinary Na/K ratio is important for BP control, even among healthy individuals.

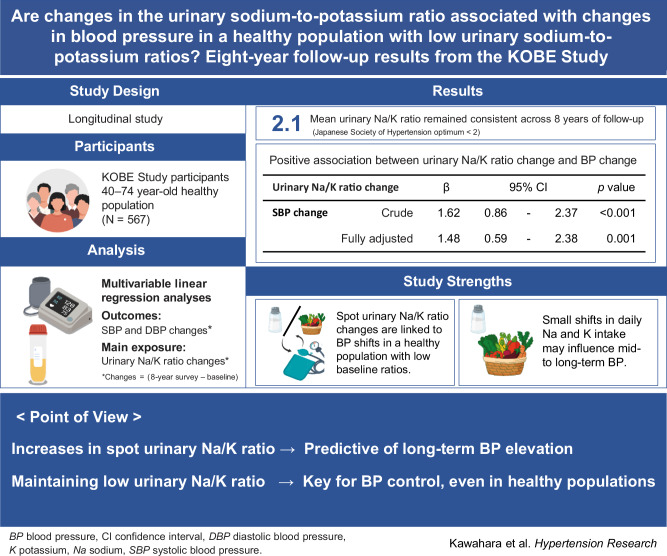

## Introduction

Cardiovascular disease (CVD) is a leading cause of death among Asian populations [1, 2], and is primarily attributed to hypertension [3, 4]. Excessive sodium (Na) intake is considered one of the main causes of hypertension in the Japanese population [5–7]. The reduction of Na intake and increased consumption of potassium (K) are recommended for the prevention of hypertension and reduction of CVD risk [8–10]. However, conventional approaches for estimating dietary intake (e.g., dietary records and 24-hour recalls) are not only laborious, but have limited accuracy in quantifying the intake of seasonings and condiments, which raises concerns about their validity [11, 12].

In recent years, the urinary Na/K ratio has received much attention as a useful marker for evaluating the dietary balance between one’s Na and K intake. As studies have shown that the urinary Na/K ratio is positively associated with blood pressure (BP) [13–15], this ratio has been proposed as a new predictor of hypertension risk [16]. In 2024, the Japanese Society of Hypertension recommended an average urinary Na/K ratio of <2 as an optimal target value for a healthy Japanese population, and <4 as a feasible target value for the general Japanese population [17].

Several studies have explored the relationships between urinary Na/K ratio changes and BP changes. For example, the Brazilian Longitudinal Study of Adult Health (ELSA-Brasil) was a long-term follow-up cohort study of higher education and research institutions in Brazil that found a positive association between changes in Na/K ratio and BP [18]. Furthermore, a study conducted in Tome City (Miyagi, Japan) reported that Na/K ratio changes were positively associated with BP changes over one year in a general population that included individuals being treated for hypertension or diabetes [19]. The Nagahama Study (Shiga, Japan) noted difficulties in assessing salt-overloading risk for longitudinal BP changes over five years using single-point measured Na/K values among community-dwelling individuals, including those with hypertension and other diseases; however, spot urine Na/K had limited utility as a prognostic marker of BP change [20]. The Shimane Community-Based Healthcare Research and Education (CoHRE) Study observed positive associations between Na/K ratio changes and BP changes in older adults aged ≥65 years (including those with hypertension) residing in Shimane Prefecture, Japan [21]. However, to the best of our knowledge, there have been no community-based, long-term follow-up studies on these associations in healthy individuals. Moreover, few studies have comprehensively evaluated the associations of changes in urinary Na levels, K levels, and Na/K ratios with changes in BP.

Therefore, this study aimed to assess the associations between changes in the urinary Na/K ratio and BP in a healthy Japanese population without hypertension, CVD, or cancer at baseline and after 8 years of follow-up.

Point of view

- Clinical relevance: An increase in the urinary Na/K ratio can predict long-term elevation in blood pressure, even in healthy individuals with near-optimal baseline ratios.
- Future direction: Further research incorporating multiple urinary measurements and home blood pressure monitoring is needed to improve the accuracy of this predictive marker.
- Consideration for the Asian population: In Asian populations, where high sodium intake is the main cause of hypertension and cardiovascular disease, measuring the urinary Na/K ratio is crucial for early prevention.

## Methods

### Study design and settings

This longitudinal study was conducted using data from the Kobe Orthopedic and Biomedical Epidemiological (KOBE) Study, a prospective population-based cohort study that was initiated in 2010 in the urban city of Kobe (Hyogo, Japan). In this study, 1117 residents were recruited between July 2010 and December 2011 through Kobe City’s municipal website and public relations initiatives. The baseline survey was conducted from July 2010 to December 2011, and the 8-year follow-up survey was conducted from May 2018 to December 2019. The meeting rooms of the Foundation for Biomedical Research and Innovation at Kobe (Hyogo, Japan) and public facilities (e.g., community centers) were used as research sites. Urine samples were collected in the morning before breakfast at these sites. The inclusion criteria were as follows: (i) aged 40–74 years; (ii) no history of malignant tumors, cerebrovascular disease, or CVD; (iii) not on medication for hypertension, diabetes, or dyslipidemia; (iv) subjectively healthy; (v) able to travel to the study site for the necessary examinations; and (vi) consent to participate in follow-up. The participants were invited to participate in an on-site survey every two years. For this study, we used data from the baseline survey (2010–2011) and an 8-year follow-up survey (2018–2019). Detailed information about the KOBE Study is available elsewhere [22–24].

### Participants

**Fig. 1:**
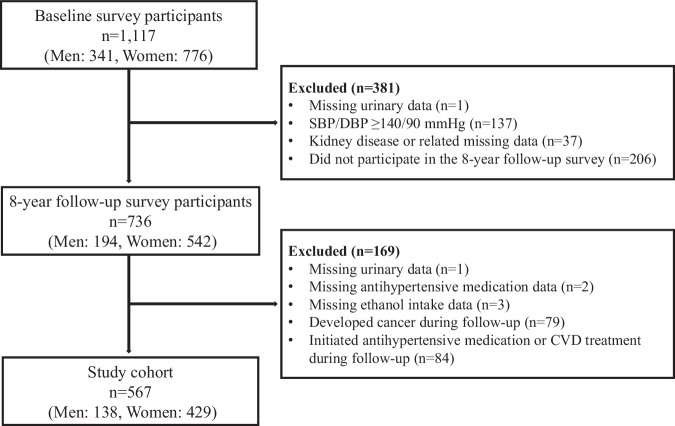
Flowchart of study participant selection. Initiation of antihypertensive medication or CVD treatment was defined as a response of “Yes” to any of the questions regarding antihypertensive medication use, stroke occurrence, or heart disease occurrence in the 8-year follow-up survey. CVD cardiovascular disease, DBP diastolic blood pressure, SBP systolic blood pressure

The flowchart of study participant selection is shown in Fig. 1. Among the 1117 baseline survey participants, we first excluded 381 participants with missing urinary data, those with systolic BP (SBP)/diastolic BP (DBP) ≥ 140/90 mmHg, those with kidney disease or related missing data, and those who did not participate in the 8-year follow-up survey. From 736 individuals who participated in the 8-year follow-up survey, we excluded 169 participants with missing urinary data, antihypertensive medication data, and ethanol intake data at the follow-up survey; those who developed cancer during follow-up; and those who initiated antihypertensive medication or CVD treatment during follow-up. Of the 84 participants who initiated antihypertensive medication or CVD treatment during follow-up, 43 had begun using antihypertensive medication, 4 were treated for stroke, 35 for heart disease, and 2 for both stroke and heart disease. The final study cohort comprised 567 participants (138 men and 429 women).

### Measurements

#### Urinary data

Spot urine samples were collected at the study site in the morning before breakfast. Fresh urine was collected in a sterile specimen cup, and immediately dispensed into a sterile tube and refrigerated before measurement. Urinary Na and K excretion levels were measured using the electrode method, and urinary creatinine (Cr) excretion levels were measured using the enzymatic method. From the spot urine samples, each participant’s urinary Na/K ratio was calculated by dividing the Na excretion level by the K excretion level. All urinary Na, K, and Cr levels were measured at a central laboratory (SRL Inc., Japan).

In addition to the spot urinary Na/K ratio, we also calculated each participant’s estimated 24-h urinary Na/K (e24hUNa/K) ratio by dividing the estimated 24-h urinary Na (e24hUNa) excretion level by the estimated 24-h urinary K (e24hUK) excretion level, which were derived from the spot urine samples based on the equations provided by Tanaka et al. [25]. We calculated e24hUNa excretion (mEq/day) from spot urinary Na and Cr values using the following formula: 21.98 × {Na (mEq/l) × estimated 24-h Cr excretion/[Cr (mg/dl) × 10]}^0.392^, where 24-h Cr excretion was predicted using the formula: −2.04 × age + 14.89 × weight (kg) + 16.14 × height (cm) – 2244.45. Next, we calculated e24hUK excretion (mEq/day) from spot urinary K and Cr values using the following formula: 7.59 × {K (mEq/l) × estimated 24-h Cr excretion/[Cr (mg/dl) × 10]}^0.431^.

The KOBE Study measured the urinary Na/K ratio only at baseline, after 6 years, and after 8 years. In order to examine the changes in urinary Na/K ratio and BP over a longer period, we used data from the 8-year follow-up survey for this analysis.

#### Blood pressure

BP measurements were taken twice using an automatic sphygmomanometer (BP-103i II; Nippon Colin, Tokyo, Japan) after participants were allowed to rest in a seated position for at least 5 min (measured by hourglass), and the mean value was recorded. In both the baseline and 8-year follow-up surveys, BP measurements were taken in the morning.

#### Covariates

Participants completed a standardized self-administered questionnaire on their medical history and lifestyle habits (e.g., smoking status, ethanol intake, years of education, and employment status) during the baseline and 8-year follow-up surveys, and trained researchers confirmed the responses through face-to-face communication. Information on the initiation of antihypertensive medication or CVD treatment was also obtained using this questionnaire. The height and weight of the participants in socks and light clothing were measured using a combined meter (U-WELL2; Elk Corp, Osaka, Japan). Body mass index (BMI) was calculated as body weight (kg) divided by the square of height (m^2^).

To assess ethanol intake, we ascertained each participant’s weekly ethanol intake based on their questionnaire responses regarding the frequency of drinking in a recent typical week and the amount consumed on each occasion, and divided this amount by seven to obtain the mean daily ethanol intake (g/day). Years of education at baseline were classified as <9, 9–12, and ≥13 years. Employment status at baseline was classified as unemployed or employed.

Blood samples were drawn from all participants after they had fasted for at least 10 h to measure low-density lipoprotein (LDL) cholesterol and hemoglobin A1c (HbA1c). Blood samples were sent to a single commissioned clinical laboratory (SRL Inc., Tokyo, Japan) for measurements. HbA1c levels were measured using high-performance liquid chromatography and expressed as National Glycohemoglobin Standardization Program units and International Federation of Clinical Chemistry and Laboratory Medicine values for analysis.

Salt taste sensitivity was assessed using Salsave, a salt-impregnated taste strip developed by Maruyama et al. [26, 27] and validated by Nishimoto et al. [28]. Participants who perceived a salty taste at a concentration of 0.6% were defined as having normal salt taste sensitivity, while those who only perceived a salty taste at higher concentrations were defined as having declined sensitivity.

The seasons in which each participant took part in the baseline survey and the 8-year follow-up survey were analyzed using the definitions specified by the Japan Meteorological Agency: spring (March to May), summer (June to August), autumn (September to November), and winter (December to February).

### Statistical analysis

The study outcomes were SBP and DBP changes, and the main independent variables of interest were urinary Na/K ratio change and e24hUNa/K ratio change. Urinary Na/K ratios using spot urine samples are typically expressed as either the spot urinary Na/K ratio or the e24hUNa/K ratio [15, 16, 29, 30]. By using these two parameters, this study aimed to comprehensively demonstrate the relationship between changes in the urinary Na/K ratio and changes in BP. The changes in urinary Na/K ratio, e24hUNa/K ratio, SBP, and DBP were calculated by subtracting the baseline values from the follow-up measurements.

First, we compared the differences in patient characteristics between the baseline and 8-year follow-up using the paired *t*-test for continuous variables and the chi-square test for categorical variables. Data were presented as mean ± standard deviation (SD) for continuous variables and as number (percentage) for categorical variables.

Next, we assessed the associations of urinary Na/K ratio change and e24hUNa/K ratio change with BP changes using multivariable linear regression models. The associations were initially analyzed using a crude unadjusted model, followed by Model 1 (adjusting for sex and age), Model 2 (adjusting for sex, age, and BMI change), and Model 3 (adjusting for sex, age, BMI change, baseline urinary Na/K ratio or e24hUNa/K ratio, baseline SBP or DBP, LDL cholesterol, HbA1c, ethanol intake change, smoking status, salt taste sensitivity, years of education, employment status, baseline survey season, and 8-year follow-up survey season).

To account for heterogeneity in the cohort, we conducted subgroup analyses in (i) a subgroup of participants with a spot urinary Na/K ratio <4 at baseline, (ii) subgroups stratified according to BMI (<18.5, 18.5–23.0, >23.0 kg/m^2^), and (iii) a subgroup of the 84 participants who were excluded from the main analysis due to initiating antihypertensive medication or CVD treatment during the 8-year follow-up period. This study adopted an upper BMI cutoff value of 23.0 kg/m^2^ based on a World Health Organization recommendation for using lower thresholds to identify “overweight” in Asian populations [31].

Furthermore, we analyzed the associations of changes in urinary Na excretion, urinary K excretion, e24hUNa excretion, and e24hUK excretion with BP changes using a crude unadjusted model and a multivariable model that adjusted for sex, age, BMI change, baseline urinary Na (or e24hUNa) excretion or K (or e24hUK) excretion, baseline SBP or DBP, LDL cholesterol, HbA1c, ethanol intake change, smoking status, salt taste sensitivity, years of education, employment status, baseline survey season, and 8-year follow-up survey season.

All statistical analyses were performed using Stata 18 (StataCorp LLC, College Station, Texas, USA), and significance was set at *p* < 0.05 (two-tailed).

## Results

Table 1 shows the characteristics of the study participants. In the overall cohort, 138 participants were men (24.3%). During the 8-year follow-up period, we observed no significant changes in the urinary Na/K ratio (2.1 ± 1.1 vs. 2.0 ± 1.1, *p* = 0.47) or the e24hUNa/K ratio (3.2 ± 0.6 vs. 3.2 ± 0.7, *p* = 0.49). The following variables were significantly lower at baseline than at the 8-year follow-up: age (57.5 ± 8.5 years vs. 65.4 ± 8.5 years, *p* < 0.001), BMI (21.3 ± 2.6 kg/m^2^ vs. 21.6 ± 2.9 kg/m^2^, *p* < 0.001), SBP/DBP (110.2/68.6 mmHg vs. 113.6/69.7 mmHg, *p* < 0.001), and HbA1c (5.5 ± 0.4% vs. 5.8 ± 0.4%, *p* < 0.001). In contrast, the following variables were significantly higher at baseline than at the 8-year follow-up: urinary Na excretion (116.0 ± 52.3 mEq/L vs. 103.2 ± 46.4 mEq/L, *p* < 0.001), urinary K excretion (64.7 ± 31.6 mEq/L vs. 58.8 ± 26.8 mEq/L, *p* < 0.001), e24hUNa excretion (143.2 ± 32.0 mEq/day vs. 138.2 ± 29.9 mEq/day, *p* < 0.001), e24hUK excretion (45.6 ± 8.5 mEq/day vs. 44.4 ± 7.7 mEq/day, *p* < 0.001), and ethanol intake (7.7 ± 15.2 g/day vs. 7.6 ± 14.1 g/day, *p* < 0.001).

**Table 1:** Characteristics of the study participants at the baseline survey and 8-year follow-up survey

	Overall (*n* = 567)		
	Baseline	8-year follow-up	*p* value
Men, *n* (%)	138 (24.3)		
Age (years)	57.5 ± 8.5	65.4 ± 8.5	<0.001
BMI (kg/m^2^)	21.3 ± 2.6	21.6 ± 2.9	<0.001
SBP (mmHg)	110.2 ± 12.7	113.6 ± 16.9	<0.001
DBP (mmHg)	68.6 ± 8.8	69.7 ± 10.4	<0.001
HbA1c (%)	5.5 ± 0.4	5.8 ± 0.4	<0.001
LDL cholesterol (mg/dL)	129.6 ± 27.8	137.5 ± 32.2	0.37
Urinary Na/K ratio	2.1 ± 1.1	2.0 ± 1.1	0.47
Urinary Na excretion (mEq/L)	116.0 ± 52.3	103.2 ± 46.4	<0.001
Urinary K excretion (mEq/L)	64.7 ± 31.6	58.8 ± 26.8	<0.001
e24hUNa/K ratio	3.2 ± 0.6	3.2 ± 0.7	0.49
e24hUNa excretion (mEq/day)	143.2 ± 32.0	138.2 ± 29.9	<0.001
e24hUK excretion (mEq/day)	45.6 ± 8.5	44.4 ± 7.7	<0.001
Ethanol intake (g/day)	7.7 ± 15.2	7.6 ± 14.1	<0.001
Current smoker, *n* (%)	23 (4.1)	21 (3.7)	0.84
Normal salt taste sensitivity, *n* (%)^a^	456 (80.4)	447^b^ (82.3)	0.42
Years of education			
<9 years	12 (2.1)	–	
9–12 years	235 (41.5)	–	
≥13 years	320 (56.4)	–	
Employment status			
Employed	485 (85.5)	–	

Data are presented as mean ± standard deviation unless stated otherwise

Continuous data were analyzed using paired *t*-tests, and categorical data were analyzed using chi-squared tests

*BMI* body mass index, *SBP* systolic blood pressure, *DBP* diastolic blood pressure, *Na* sodium, *K* potassium, *e24hUK* estimated 24-h urinary potassium, *e24hUNa* estimated 24-h urinary sodium, *e24hUNa/K* estimated 24-h urinary sodium/potassium, *HbA1c* hemoglobin A1c, *LDL* low-density lipoprotein

^a^Normal salt taste sensitivity: salty taste perception at ≤0.6% assessed using Salsave

^b^Denominator: *n* = 543

Table 2 shows the associations of urinary Na/K ratio change and e24hUNa/K ratio change with BP changes during the 8-year follow-up period. Both urinary Na/K ratio change and e24hUNa/K ratio change showed significant and positive associations with BP changes in all models. When analyzed using Model 3, the β coefficients were 1.48 (*p* = 0.001) for urinary Na/K ratio change and SBP change, 2.26 (*p* = 0.004) for e24hUNa/K ratio change and SBP change, 0.76 (*p* = 0.004) for urinary Na/K ratio change and DBP change, and 1.06 (*p* = 0.020) for e24hUNa/K ratio change and DBP change.

**Table 2:** Associations of urinary Na/K ratio change and e24hUNa/K ratio change with BP changes

		Overall (*n* = 567)		
		β	95% CI	*p* value
Urinary Na/K ratio change				
SBP change	Crude	1.62	0.86 − 2.37	<0.001
	Model 1	1.74	0.99 − 2.49	<0.001
	Model 2	1.57	0.84 − 2.30	<0.001
	Model 3	1.48	0.59 − 2.38	0.001
DBP change	Crude	1.11	0.67 − 1.55	<0.001
	Model 1	1.10	0.66 − 1.54	<0.001
	Model 2	1.01	0.57 − 1.44	<0.001
	Model 3	0.76	0.24 − 1.29	0.004
e24hUNa/K ratio change				
SBP change	Crude	2.74	1.44 − 4.04	<0.001
	Model 1	2.93	1.64 − 4.21	<0.001
	Model 2	2.54	1.28 − 3.80	<0.001
	Model 3	2.26	0.72 − 3.79	0.004
DBP change	Crude	1.76	1.00 − 2.51	<0.001
	Model 1	1.74	0.98 − 2.50	<0.001
	Model 2	1.53	0.78 − 2.28	<0.001
	Model 3	1.06	0.17 − 1.96	0.020

*BP* blood pressure, *CI* confidence interval, *SBP* systolic blood pressure*, DBP* diastolic blood pressure, *e24hUNa/K* estimated 24-h urinary sodium/potassium, *Na* sodium, *K* potassium

Model 1: Adjusted for sex and age

Model 2: Adjusted for sex, age, and body mass index change

Model 3: Adjusted for sex, age, body mass index change, baseline urinary Na/K ratio or e24hUNa/K ratio, baseline SBP or DBP, low-density lipoprotein cholesterol, hemoglobin A1c, ethanol intake change, smoking status, salt taste sensitivity, years of education, employment status, baseline survey season, and 8-year follow-up survey season

In the subgroup analysis of 537 participants with a spot urinary Na/K ratio <4 at baseline, the associations of urinary Na/K ratio change and e24hUNa/K ratio change with BP changes remained significant and positive in all models (Supplementary Table S1). Participants with BMI < 18.5 kg/m² showed minimal changes in BP and almost no change in the urinary Na/K ratio (Supplementary Table S2). Next, significant and positive associations were observed between urinary Na/K ratio change and BP changes in Model 3 for participants with BMI of 18.5–23.0 kg/m^2^ and >23.0 kg/m^2^, but not for those with BMI < 18.5 kg/m^2^ (Supplementary Table S3). Similarly, e24hUNa/K ratio change showed significant and positive associations with BP changes in Model 3 for participants with BMI > 23.0 kg/m^2^; however, e24hUNa/K ratio change was only significantly associated with SBP in Model 3 for participants with BMI of 18.5–23.0 kg/m^2^. Supplementary Table S4 shows the associations of urinary Na/K ratio change and e24hUNa/K ratio change with BP changes in the subgroup of 84 participants who were excluded due to initiating antihypertensive medication or CVD treatment during the 8-year follow-up period. In all models, no positive associations were observed among these participants.

The associations of urinary Na excretion change, urinary K excretion change, e24hUNa excretion change, and e24hUK excretion change with BP changes are shown in Table 3. In the adjusted models, urinary Na excretion change and e24hUK excretion change were not associated with BP changes. Urinary K excretion change was significantly associated with SBP change, but not with DBP change. In contrast, e24hUNa excretion change was significantly associated with both SBP and DBP changes.

**Table 3:** Associations of urinary Na excretion change, urinary K excretion change, e24hUNa excretion change, and e24hUK excretion change with BP changes

		Overall (*n* = 567)		
	Model	β	95% CI	*p* value
Urinary Na excretion change				
SBP change	Crude	0.02	−0.00 − 0.04	0.118
	Adjusted^a^	0.01	−0.01 − 0.04	0.303
DBP change	Crude	0.01	0.00 − 0.02	0.019
	Adjusted^a^	0.01	−0.00 − 0.02	0.160
Urinary K excretion change				
SBP change	Crude	−0.04	−0.07 − −0.01	0.016
	Adjusted^a^	−0.04	−0.07 − 0.00	0.069
DBP change	Crude	−0.02	−0.03 − −0.00	0.046
	Adjusted^a^	−0.01	−0.03 − 0.01	0.356
e24hUNa excretion change				
SBP change	Crude	0.04	0.01 − 0.07	0.004
	Adjusted^b^	0.05	0.01 − 0.08	0.012
DBP change	Crude	0.03	0.01 − 0.04	0.004
	Adjusted^b^	0.02	0.00 − 0.04	0.047
e24hUK excretion change				
SBP change	Crude	−0.10	−0.23 − 0.02	0.100
	Adjusted^b^	−0.03	−0.17 − 0.11	0.664
DBP change	Crude	−0.07	−0.14 − 0.00	0.051
	Adjusted^b^	−0.02	−0.10 − 0.06	0.639

*BP* blood pressure, *CI* confidence interval, *SBP* systolic blood pressure, *DBP* diastolic blood pressure, *e24hUK* estimated 24-h urinary potassium, *e24hUNa* estimated 24-h urinary sodium, *Na* sodium, *K* potassium

^a^Adjusted for sex, age, body mass index change, baseline urinary Na excretion or urinary K excretion, baseline SBP or DBP, low-density lipoprotein cholesterol, hemoglobin A1c, ethanol intake change, smoking status, salt taste sensitivity, years of education, employment status, baseline survey season, and 8-year follow-up survey season

^b^Adjusted for sex, age, body mass index change, baseline e24hUNa excretion or e24hUK excretion, baseline SBP or DBP, low-density lipoprotein cholesterol, hemoglobin A1c, ethanol intake change, smoking status, salt taste sensitivity, years of education, employment status, baseline survey season, and 8-year follow-up survey season

## Discussion

This longitudinal cohort study showed that changes in the spot urinary Na/K ratio and e24hUNa/K ratio were positively associated with changes in both SBP and DBP in a healthy Japanese urban population with no history of treatment for hypertension or CVD over 8 years of follow-up.

The mean spot urinary Na/K ratio in our study cohort was 2.1 at baseline, which was lower than those reported in previous studies [19–21, 32]. Accordingly, our analyses showed that urinary Na/K ratio changes were associated with BP changes in a population with a near-optimal ratio for a healthy Japanese population [17]. In contrast, the spot urinary Na/K ratios were relatively high in the Tome City study (mean ± SD: 5.4 ± 3.0) [19], the Nagahama study (mean ± SD: 3.2 ± 1.8) [20], and the Shimane CoHRE study (median: 2.5, interquartile range: 1.7–3.4) [21]. The low baseline urine Na/K ratio compared to previous studies may have been influenced by differences in the characteristics of the study population. First, the KOBE study is a population-based cohort study investigating risk factors for a decline in quality of life or CVD. Participants were healthy community residents of Kobe City, recruited by the Kobe Municipal Government’s Public Relations Office through advertisements distributed in public facilities. This ensured that participants were highly health-conscious volunteers. Furthermore, this study only included healthier residents, and those with a history of malignant tumors, cerebrovascular disease, CVD or who were taking medication for hypertension, diabetes or dyslipidaemia were excluded. Participants who started taking antihypertensive medication or CVD treatment during the eight-year follow-up period were also excluded. In contrast, The Tome City study collected data through specific health check-up conducted in Tome City, Miyagi. The majority of these participants had National Health Insurance. The Tome City study included higher proportions of antihypertensive users (39.4–41.7%) and heart disease patients (6.4–7.7%) [19]. The Nagahama study also collected data through specific health check-up conducted in Nagahama City, Shiga. The Nagahama study included higher proportions of antihypertensive users (16.0%) and hypertensives (30.3%) excluding history of CVD and insulin therapy or hemodialysis, pacemaker implantation [20]. The Shimane CoHRE study collected data through health check-up conducted in some cities and towns, Shimane. Participants in the Shimane CoHRE study were aged ≥65 years and included a high proportion of antihypertensive users (36.3%) [21]. Second, our study cohort comprised residents of Kobe City in western Japan, which has been reported by the INTERSALT Study to have lower Na excretion than other regions in eastern Japan [5]. Furthermore, ~70% of our participants were women, which was higher than in previous reports [33, 34]; this difference in sex distribution may have contributed to the lower urinary Na/K ratios in our cohort.

Next, the participants in our study had normal ranges in BP and BMI (SBP: 110.2 ± 12.7 mmHg, DBP: 68.6 ± 8.8 mmHg, and BMI: 21.3 ± 2.6 kg/m²). The mean SBP in our study cohort was considerably lower than in ELSA-Brasil (mean ± SD: 121 ± 13 mmHg for men and 111 ± 12 mmHg for women) [18], the Tome City study (mean ± SD: 132.1 ± 17.9 mmHg) [19], the Nagahama study (mean ± SD: 124 ± 17 mmHg) [20] and the Shimane CoHRE study (median: 128 mmHg, interquartile range: 116–137 mmHg) [21]. Moreover, the mean BMI in our study cohort was lower than in ELSA-Brasil (mean ± SD: 25.9 ± 3.6 kg/m^2^ for men and 25.4 ± 4.1 kg/m^2^ for women) [18], the Tome City study (mean ± SD: 23.8 ± 3.6 kg/m^2^) [19], the Nagahama study (mean ± SD: 22.3 ± 3.2 kg/m^2^) [20] and the Shimane CoHRE study (median: 22.0 kg/m^2^, interquartile range: 19.9–23.8 kg/m^2^) [21]. Thus, our analysis of KOBE Study data demonstrated that increases in the urinary Na/K ratio were significantly and consistently associated with BP elevation in a healthy population that can be considered to have a low risk of hypertension and CVD. Furthermore, our analyses did not find any significant associations between urinary Na/K ratio change and BP changes in participants with BMI < 18.5 kg/m^2^. As this group experienced almost no changes in BP and urinary Na/K ratio during follow-up, it may have been difficult to demonstrate a relationship between these parameters.

To our knowledge, this study is the first to report that changes in the spot urinary Na/K ratio were associated with BP changes over an 8-year period in a healthy population that did not subsequently undergo treatment for hypertension or CVD. In contrast, our subgroup analysis of the 84 individuals who initiated antihypertensive medication or CVD treatment during follow-up found no significant associations between changes in the urinary Na/K ratio and BP, which could be attributed to their successful control of BP through the use of antihypertensive medications. The participants in the Tome City study [19] and the Shimane CoHRE study [21] included individuals who were undergoing treatment for hypertension or diabetes, and stratification was based solely on the use or non-use of antihypertensive medications at baseline without accounting for the subsequent initiation of new medications.

Our study also found that e24hUNa/K ratios were associated with greater BP changes than spot urinary Na/K ratios. However, urinary Cr is required to estimate the e24hUNa and e24hUK levels, which hinders rapid evaluations. Therefore, using a simple Na/K ratio can facilitate prompt assessments without requiring additional laboratory measurements [29, 30]. The present study found no clear association between changes in urinary K excretion and BP changes, which may be because approximately 10% of ingested K is excreted in the feces [35]. Due to the lack of hypertensive individuals, our study cohort demonstrated a near-optimal urinary Na/K ratio as recommended by the Japanese Society of Hypertension [17]. In spite of this, we observed associations between the urinary Na/K ratio and long-term increases in BP, which highlights the importance of maintaining a low ratio even in individuals who are healthy and normotensive. Based on these findings, the inclusion of urinary Na/K ratio changes to conventional assessments of urinary Na and K excretion may provide a more accurate prediction of hypertension risk.

Changes in the urinary Na/K ratios, in Na excretion, and in K excretion were reduced at the 8-year follow-up compared to baseline. This may be due to a decline in food intake caused by ageing or a reduction in salt intake, but further studies are needed to explore this relationship.

This study has several limitations. First, the urinary Na/K ratio was measured at only two time points (baseline and 8 years later), and changes during the intervening period were not assessed. Second, the urinary Na/K ratio was measured only once at each time point. However, single measurements of the spot urinary Na/K ratio have been used for population-level assessments [25, 29, 36], and may therefore have applications in monitoring long-term changes in populations. Further research with multiple measurements is required. Third, BP measurements were limited to those taken at the study site. The inclusion of home BP monitoring could enable a more precise and robust investigation of the associations between changes in urinary Na/K ratio and BP. Finally, this study lacked pregnancy information, which may have influenced the results. However, all female participants were aged ≥40 years, with a mean ± SD age of 57.2 ± 8.4 years at baseline. Consequently, the number of women who could be pregnant was considered to be extremely low, and were unlikely to have a substantial impact on our results.

### Perspective of Asia

CVD remains a leading cause of mortality in Asia, primarily due to hypertension [1, 2]. The diets of East Asians are characterized by high sodium intake from seasonings, posing a critical public health challenge [5–7]. In Asian populations where these dietary habits are the main cause of disease, the urinary Na/K ratio is an important metric for early prevention. Therefore, the JSH 2024 Consensus Statement on Urine Sodium-to-Potassium Ratio recently recommended a target ratio of less than 2.0 for healthy individuals [17]. Our study showed that, even in a Japanese cohort with near-optimal baseline ratios, an increase in the Na/K ratio over time is directly associated with an increase in BP.

## Conclusion

In conclusion, increases in spot urinary Na/K ratios were predictive of long-term BP elevation in a healthy, normotensive Japanese population with near-optimal urinary Na/K ratios at baseline. These findings indicate the need to regularly monitor and maintain low urinary Na/K ratios as a means to control BP, even in healthy populations.

## Supplementary information

Supplementary Table.S1

Supplementary Table.S2

Supplementary Table.S3

Supplementary Table.S4
