# Supplementary material for: Are changes in the urinary sodium-to-potassium ratio associated with changes in blood pressure in a healthy population with low urinary sodium-to-potassium ratios? Eight-year follow-up results from the KOBE Study
Source: Hypertens Res. 2026 Apr 13;49(6):1878–87. doi: 10.1038/s41440-026-02621-9 (PMC13236578; doi:10.1038/s41440-026-02621-9)
Supplement: Supplementary file 1 — Supplementary Table.S1 [file 41440_2026_2621_MOESM1_ESM.pdf]

Supplementary Table S1. Subgroup analysis: Associations of urinary Na/K ratio change and e24hUNa/K ratio change with BP changes among participants with a spot urinary Na/K ratio <4 at baseline

		Overall (n=537)				
		β	95% CI			*p* value
**Urinary Na/K ratio change**						
SBP change	Crude	1.90	1.01	−	2.78	<0.001
	Model 1	1.99	1.11	−	2.87	<0.001
	Model 2	1.75	0.89	−	2.61	<0.001
	Model 3	1.60	0.66	−	2.54	0.001
DBP change	Crude	1.10	0.58	−	1.62	<0.001
	Model 1	1.07	0.55	−	1.59	<0.001
	Model 2	0.94	0.43	−	1.45	<0.001
	Model 3	0.79	0.25	−	1.34	0.004
**e24hUNa/K ratio change**						
SBP change	Crude	3.05	1.61	−	4.49	<0.001
	Model 1	3.19	1.76	−	4.62	<0.001
	Model 2	2.71	1.30	−	4.11	<0.001
	Model 3	2.48	0.89	−	4.07	0.002
DBP change	Crude	1.67	0.83	−	2.51	<0.001
	Model 1	1.62	0.77	−	2.47	<0.001
	Model 2	1.36	0.52	−	2.19	0.002
	Model 3	1.09	0.17	−	2.02	0.021

BP blood pressure, *CI* confidence interval, *SBP* systolic blood pressure, *DBP* diastolic blood pressure, *Na* sodium, *K* potassium, *e24hUNa/K* estimated 24-h urinary sodium/potassium.
Model 1: Adjusted for sex and age.
Model 2: Adjusted for sex, age, and body mass index change.
Model 3: Adjusted for sex, age, body mass index change, baseline urinary Na/K ratio or e24hUNa/K ratio, baseline SBP or DBP, low-density lipoprotein cholesterol, hemoglobin A1c, ethanol intake change, smoking status, salt taste sensitivity, years of education, employment status, baseline survey season, and 8-year follow-up survey season.
