# Supplementary material for: Are changes in the urinary sodium-to-potassium ratio associated with changes in blood pressure in a healthy population with low urinary sodium-to-potassium ratios? Eight-year follow-up results from the KOBE Study
Source: Hypertens Res. 2026 Apr 13;49(6):1878–87. doi: 10.1038/s41440-026-02621-9 (PMC13236578; doi:10.1038/s41440-026-02621-9)
Supplement: Supplementary file 2 — Supplementary Table.S2 [file 41440_2026_2621_MOESM2_ESM.pdf]

Supplementary Table S2. Characteristics of the study participants at the baseline survey and 8-year follow-up survey stratified by BMI

	BMI Status (n=567)					
	<18.5 kg/m^2 (n=80)		18.5–23.0 kg/m^2 (n=355)		>23.0 kg/m^2 (n=132)	
	Baseline	8-year follow-up	Baseline	8-year follow-up	Baseline	8-year follow-up
Men, n (%)	3 (3.8)		82 (23.1)		66 (40.2)	
Age (years)	55.4 ± 9.3	63.3 ± 9.4	57.6 ± 8.3	65.5 ± 8.3	58.3 ± 8.4	66.2 ± 8.4
BMI (kg/m^2)	17.6 ± 0.9	17.8 ± 1.3	20.8 ± 1.2	21.1 ± 1.7	24.8 ± 1.7	25.2 ± 2.2
SBP (mmHg)	101.6 ± 12.1	103.5 ± 18.1	109.7 ± 12.3	113.1 ± 16.0	116.9 ± 10.8	121.2 ± 15.2
DBP (mmHg)	62.7 ± 8.4	62.9 ± 10.8	68.0 ± 8.4	69.3 ± 10.0	73.8 ± 7.3	75.1 ± 8.4
HbA1c (%)	5.4 ± 0.3	5.7 ± 0.3	5.5 ± 0.3	5.8 ± 0.4	5.6 ± 0.4	5.9 ± 0.5
LDL cholesterol (mg/dL)	124.1 ± 26.0	135.1 ± 27.0	129.0 ± 28.1	136.5 ± 34.4	134.6 ± 27.3	141.5 ± 28.4
Urinary Na/K ratio	1.8 ± 1.0	1.8 ± 0.9	2.1 ± 1.1	2.0 ± 1.1	2.2 ± 1.1	2.2 ± 1.2
Urinary Na excretion (mEq/L)	106.0 ± 50.1	92.4 ± 44.5	114.5 ± 51.2	101.7 ± 44.6	126.2 ± 55.1	113.9 ± 50.3
Urinary K excretion (mEq/L)	66.0 ± 31.1	56.9 ± 27.2	64.3 ± 32.2	59.1 ± 26.5	65.1 ± 30.5	59.2 ± 27.5
e24hUNa/K ratio	3.0 ± 0.7	3.1 ± 0.6	3.2 ± 0.6	3.1 ± 0.7	3.2 ± 0.6	3.3 ± 0.7
e24hUNa excretion (mEq/day)	125.9 ± 30.6	127.0 ± 27.0	142.2 ± 29.9	136.9 ± 29.1	156.3 ± 32.9	148.6 ± 30.7
e24hUK excretion (mEq/day)	41.7 ± 7.3	41.9 ± 7.5	45.3 ± 8.2	44.3 ± 7.6	48.8 ± 8.9	46.1 ± 7.8
Ethanol intake (g/day)	3.5 ± 7.5	5.1 ± 10.2	7.5 ± 14.2	7.8 ± 14.3	10.7 ± 19.9	8.7 ± 15.5
Current smoker, n (%)	0 (0.0)	0 (0.0)	13 (3.7)	11 (3.1)	10 (7.6)	10 (7.6)
Normal salt taste sensitivity, n (%)[a]	65 (81.2)	71[b] (89.9)	289 (81.4)	273[c] (81.0)	102 (77.3)	103[d] (81.1)
Years of education						
<9 years	0 (0.0)	−	9 (2.5)	−	3 (2.3)	−
9–12 years	28 (35.0)	−	150 (42.3)	−	57 (43.2)	−
≥13 years	52 (65.0)	−	196 (55.2)	−	72 (54.5)	−
Employment status						
Employed	72 (90.0)	−	303 (85.4)	−	110 (83.3)	−

Data are presented as mean ± standard deviation unless stated otherwise.

Continuous data were analyzed using paired *t*-tests, and categorical data were analyzed using chi-squared tests.

BMI body mass index, *SBP* systolic blood pressure, *DBP* diastolic blood pressure, *Na* sodium, *K* potassium, *e24hUK* estimated 24-h urinary potassium, *e24hUNa* estimated 24-h urinary sodium, *e24hUNa/K* estimated 24-h urinary sodium/potassium, *HbA1c* hemoglobin A1c, *LDL* low-density lipoprotein.

[a] Normal salt taste sensitivity: salty taste perception at ≤0.6% assessed using Salsave.

[b] Denominator: n = 79

[c] Denominator: n = 337

[d] Denominator: n = 127
