# Supplementary material for: Are changes in the urinary sodium-to-potassium ratio associated with changes in blood pressure in a healthy population with low urinary sodium-to-potassium ratios? Eight-year follow-up results from the KOBE Study
Source: Hypertens Res. 2026 Apr 13;49(6):1878–87. doi: 10.1038/s41440-026-02621-9 (PMC13236578; doi:10.1038/s41440-026-02621-9)
Supplement: Supplementary file 3 — Supplementary Table.S3 [file 41440_2026_2621_MOESM3_ESM.pdf]

Supplementary Table S3. Subgroup analysis: Associations of urinary Na/K ratio change and e24hUNa/K ratio change with BP changes stratified by BMI

		BMI Status (n=567)								
		<18.5 kg/m^2 (n=80)			18.5–23.0 kg/m^2 (n=355)			>23.0 kg/m^2 (n=132)		
		β	95% CI	*p* value	β	95% CI	*p* value	β	95% CI	*p* value
**Urinary Na/K ratio change**										
SBP change	Crude	0.51	-1.74 – 2.76	0.65	1.77	0.81 – 2.74	<0.001	1.78	0.31 – 3.24	0.02
	Model 1	0.63	-1.66 – 2.91	0.59	2.00	1.04 – 2.96	<0.001	1.90	0.44 – 3.37	0.01
	Model 2	1.20	-2.14 – 4.54	0.48	1.76	0.61 – 2.91	0.003	2.27	0.48 – 4.07	0.01
	Model 3	0.13	-3.48 – 3.73	0.94	1.34	0.20 – 2.48	0.02	2.14	0.27 – 4.01	0.03
DBP change	Crude	0.58	-0.65 – 1.81	0.35	1.10	0.54 – 1.65	<0.001	1.41	0.50 – 2.31	0.003
	Model 1	0.71	-0.59 – 1.98	0.27	1.11	0.55 – 1.67	<0.001	1.39	0.48 – 2.31	0.003
	Model 2	0.67	-1.18 – 2.53	0.47	0.83	0.16 – 1.50	0.02	1.52	0.39 – 2.65	0.009
	Model 3	-0.23	-2.13 – 1.67	0.81	0.60	-0.07 – 1.27	0.08	1.23	0.15 – 2.30	0.03
**e24hUNa/K ratio change**										
SBP change	Crude	0.71	-2.65 – 4.06	0.68	3.43	1.77 – 5.10	<0.001	2.43	-0.28 – 5.13	0.08
	Model 1	0.89	-2.49 – 4.27	0.60	3.79	2.14 – 5.44	<0.001	2.67	-0.06 – 5.39	0.06
	Model 2	1.26	-3.57 – 6.08	0.61	3.38	1.40 – 5.36	0.001	3.10	-0.10 – 6.30	0.06
	Model 3	-0.94	-6.09 – 4.21	0.72	2.41	0.47 – 4.35	0.02	3.05	-0.26 – 6.35	0.07
DBP change	Crude	0.63	-1.21 – 2.47	0.50	1.92	0.95 – 2.88	<0.001	2.23	0.55 – 3.91	0.01
	Model 1	0.79	-1.10 – 2.67	0.41	1.94	0.97 – 2.91	<0.001	2.22	0.51 – 3.92	0.01
	Model 2	0.84	-1.85 – 3.53	0.54	1.42	0.27 – 2.58	0.02	2.37	0.37 – 4.37	0.02
	Model 3	-0.71	-3.44 – 2.03	0.61	0.88	-0.27 – 2.02	0.13	1.94	0.08 – 3.80	0.04

BMI body mass index, *BP* blood pressure, *CI* confidence interval, *SBP* systolic blood pressure, *DBP* diastolic blood pressure, *Na* sodium, *K* potassium, *e24hUNa/K* estimated 24-h urinary sodium/potassium,.

Model 1: Adjusted for sex and age.

Model 2: Adjusted for sex, age, and baseline urinary Na/K ratio or e24hUNa/K ratio.

Model 3: Adjusted for sex, age, baseline urinary Na/K ratio or e24hUNa/K ratio, baseline SBP or DBP, low-density lipoprotein cholesterol, hemoglobin A1c, ethanol intake change, smoking status, salt taste sensitivity, years of education, employment status, baseline survey season, and 8-year follow-up survey season.
