# Supplementary material for: Are changes in the urinary sodium-to-potassium ratio associated with changes in blood pressure in a healthy population with low urinary sodium-to-potassium ratios? Eight-year follow-up results from the KOBE Study
Source: Hypertens Res. 2026 Apr 13;49(6):1878–87. doi: 10.1038/s41440-026-02621-9 (PMC13236578; doi:10.1038/s41440-026-02621-9)
Supplement: Supplementary file 4 — Supplementary Table.S4 [file 41440_2026_2621_MOESM4_ESM.pdf]

Supplementary Table S4. Subgroup analysis: Associations of urinary Na/K ratio change and e24hUNa/K ratio change with BP changes among participants who had initiated antihypertensive medication or CVD treatment during the 8-year follow-up period

		Initiation of antihypertensive medication or CVD treatment									
		No (Main Analysis) (n=567)					Yes (n=84)				
		β	95% CI			*p* value	β	95% CI			*p* value
**Urinary Na/K ratio change**											
SBP change	Crude	1.62	0.86	–	2.37	<0.001	1.98	-1.33	–	5.29	0.24
	Model 1	1.74	0.99	–	2.49	<0.001	1.65	-1.89	–	5.18	0.36
	Model 2	1.57	0.84	–	2.30	<0.001	0.39	-3.17	–	3.95	0.83
	Model 3	1.48	0.59	–	2.38	0.001	0.68	-3.41	–	4.78	0.74
DBP change	Crude	1.11	0.67	–	1.55	<0.001	1.12	-0.95	–	3.19	0.28
	Model 1	1.10	0.66	–	1.54	<0.001	0.59	-1.55	–	2.72	0.59
	Model 2	1.01	0.57	–	1.44	<0.001	-0.05	-2.22	–	2.13	0.97
	Model 3	0.76	0.24	–	1.29	0.004	0.73	-1.66	–	3.12	0.55
**e24hUNa/K ratio change**											
SBP change	Crude	2.74	1.44	–	4.04	<0.001	3.58	-1.84	–	9.01	0.19
	Model 1	2.93	1.64	–	4.21	<0.001	3.14	-2.55	–	8.82	0.28
	Model 2	2.54	1.28	–	3.80	<0.001	1.41	-4.26	–	7.07	0.62
	Model 3	2.26	0.72	–	3.79	0.004	1.73	-4.75	–	8.22	0.60
DBP change	Crude	1.76	1.00	–	2.51	<0.001	1.92	-1.46	–	5.31	0.26
	Model 1	1.74	0.98	–	2.50	<0.001	1.27	-2.16	–	4.70	0.46
	Model 2	1.53	0.78	–	2.28	<0.001	0.41	-3.06	–	3.88	0.82
	Model 3	1.06	0.17	–	1.96	0.020	1.17	-2.58	–	4.91	0.54

BP blood pressure, *CI* confidence interval, *CVD* cardiovascular disease, *SBP* systolic blood pressure, *DBP* diastolic blood pressure, *Na* sodium, *K* potassium, *e24hUNa/K* estimated 24-h urinary sodium/potassium.

Model 1: Adjusted for sex and age.

Model 2: Adjusted for sex, age, and body mass index change.

Model 3: Adjusted for sex, age, body mass index change, baseline urinary Na/K ratio or e24hUNa/K ratio, baseline SBP or DBP, low-density lipoprotein cholesterol, hemoglobin A1c, ethanol intake change, smoking status, salt taste sensitivity, years of education, employment status, baseline survey season, and 8-year follow-up survey season.
